# Erythropoietin/erythropoietin-receptor system is involved in angiogenesis in human hepatocellular carcinoma

**DOI:** 10.1111/j.1365-2559.2007.02654.x

**Published:** 2007-04-01

**Authors:** D Ribatti, A Marzullo, A Gentile, V Longo, B Nico, A Vacca, F Dammacco

**Affiliations:** Department of Human Anatomy and Histology, University of Bari Medical School Bari, Italy; 1 Department of Pathology and Genetics, University of Bari Medical School Bari, Italy; 2 Department of Internal Medicine and Clinical Oncology, University of Bari Medical School Bari, Italy

**Keywords:** angiogenesis, erythropoietin, erythropoietin receptor, hepatocellular carcinoma

## Abstract

Ribatti D, Marzullo A, Gentile A, Longo V, Nico B, Vacca A & Dammacco F (2007) *Histopathology* **50,** 591–596 **Erythropoietin/erythropoietin-receptor system is involved in angiogenesis in human hepatocellular carcinoma**

**Aims:**

To correlate microvascular density and erythropoietin (Epo)/Epo-receptor (EpoR) expression in endothelial and tumour cells with histopathological type in hepatocellular carcinoma (HCC).

**Methods and results:**

Specimens of primary HCC obtained from 50 patients who had undergone curative hepatectomy were investigated immunohistochemically by using anti-CD31, anti-Epo and anti-EpoR antibodies. Poorly differentiated HCC had a higher degree of vascularization than other stages and Epo/EpoR expression in both tumour and endothelial cells increased in parallel with grade of malignancy and was highly correlated with the extent of angiogenesis.

**Conclusions:**

Epo/EpoR levels correlate with angiogenesis and progression of patients with HCC and these findings suggest the presence of a loop in the Epo/EpoR system, i.e. Epo is secreted by hepatic tumour cells and it affects vascular endothelial cells via its receptors and promotes angiogenesis in a paracrine manner. It is thus suggested that Epo is an important factor in hepatic tumour angiogenesis. Understanding the mechanisms of HCC angiogenesis provides a basis for a rational approach to the development of antiangiogenic therapy in patients with hepatic cancer.

## Introduction

Erythropoietin (Epo) is a low-molecular-weight glycoprotein hormone stimulator of erythropoiesis produced in the fetal liver and subsequently in the adult kidney.^1^ Epo exerts its action through its specific receptor (EpoR), a member of the cytokine receptor superfamily, which is mainly expressed on erythroid colony-forming units.^2^

Epo is a pleiotropic cytokine that exerts diverse biological effects in many non-haematopoietic tissues. There is increasing evidence suggesting a wider biological role for Epo/EpoR unrelated to erythropoiesis. Angiogenesis, the process by which new blood vessels arise from pre-existing ones, has been shown to be one of the extrahaematopoietic functions of Epo.^3^ The precise role of Epo in angiogenesis has not been clarified, although many critical functions of Epo have been reported. Endothelial cells from some sources express EpoR.^4^ Moreover, Epo induces endothelial cell proliferation and migration^4^–^6^ and has been shown to stimulate angiogenesis in rat aortic rings *in vitro.*^7^ We have demonstrated that recombinant human Epo (rhEpo) induces a proangiogenic phenotype in human endothelial cells.^8^ This phenotype includes both early (i.e. increase in cell proliferation and matrix metalloproteinase-2 production) and late (differentiation into vascular tubes) angiogenic events. Accordingly, endothelial cells express EpoR that bind to Jak2 and induce its transient activation after rhEpo exposure. In the chick embryo chorioallantoic membrane (CAM) assay, the angiogenic activity of rhEpo is quantitatively and qualitatively similar to that exerted by fibroblast growth factor-2 (FGF-2) and CAM endothelial cells express EpoR that colocalizes with factor VIII-related antigen positivity. Jaquet *et al*.^9^ found the angiogenic potential of Epo to be similar to that of vascular endothelial growth factor (VEGF) when stimulating human adult myocardial endothelial cells.

The potential role of Epo in angiogenesis may be considered as a subsidiary of its possible function in improving overall tissue oxygenation and of its antiapoptotic role. The expression of EpoR in tumour vascular endothelium suggests that Epo may affect the tumour microenvironment, perhaps by stimulating tumour angiogenesis.^3^

Experimental and clinical data indicate that tumour progression in human hepatocellular carcinoma (HCC) is associated with angiogenesis and that an increase in microvascular density is associated with a poor prognosis.^10^ It is becoming increasingly evident that agents which interfere with blood vessel formation also block tumour progression. Accordingly, antiangiogenic tumour therapy has attracted much interest in preclinical and clinical assessments of HCC.^11^

In this study, we investigated immunohistochemical expression of Epo/EpoR in HCC and correlated this with CD31 expression in vascular endothelial cells with the aim of establishing a potential role for Epo in angiogenesis in HCC.

## Materials and methods

### Patient population and tumours

Fifty patients (26 males and 24 females, mean age 61 ± 5 years, range 22–75) with a singular nodule of HCC who had undergone curative hepatectomy from January 1996 to December 2002 at the Hepato-Biliary-Pancreatic Surgery Division, University of Bari Medical School, were included in the study. Patients who had had a previous hepatectomy or hepatic arterial chemoembolization were excluded. All patients had liver cirrhosis, they were not alcoholic and tested negative for hepatitis B surface antigen (HBsAg) and for antibodies to hepatitis C (anti-HCV). HCC tissues and surrounding cirrhotic liver tissues were examined. Liver biopsy samples from 10 patients with minimal reactive changes (normal liver) or with histological changes of non-immune-mediated cholestasis (mild portal inflammation and/or ductular hyperplasia) were also studied. Biopsies were obtained during abdominal surgery for uncomplicated cholelithiasis and all these patients were negative for HBsAg, anti-HCV and autoantibodies. Tumours were graded as well differentiated (tumour size maximum diameter = 30.0 ± 15.4 mm), moderately differentiated (tumour size maximum diameter = 51.5 ± 24.6 mm) or poorly differentiated (tumour size maximum diameter = 82.4 ± 42.3 mm). HCC develops and progresses from a small-sized, well-differentiated histotype with no developed blood vessels to a larger and moderately differentiated or poorly differentiated one with characteristic hypervascularity during the differentiation process.^12^ Tissue samples were fixed in 10% buffered formalin and embedded in paraffin according to standard procedures. Sections (4 µm thick) were cut and mounted on glass slides.

### Immunohistochemistry

A murine monoclonal antibody (MAb) against the endothelial cell marker CD31, a more sensitive marker for endothelial cells than factor VIII antigen^13^ (MAb 1A10; DakoCytomation, Glostrup, Denmark) and two rabbit polyclonal antibodies against Epo and EpoR (N19 and C20; Santa Cruz Biotechnology, Santa Cruz, CA, USA) were used. The anti-Epo and the anti-EpoR antibodies are two affinity-purified rabbit polyclonal antibodies raised against a peptide mapping at the N-terminus of Epo and, respectively, against amino acids 21–214 mapping near the N-terminus of EpoR of human origin. Briefly, sections were collected on 3-amino-propyl-triethoxysilane-coated slides, deparaffinized by the xylene–ethanol sequence, rehydrated in a graded ethanol scale and in Tris-buffered saline (TBS, pH 7.6) and incubated overnight at 4°C with the MAb 1A10 (1 : 25 in TBS) and the polyclonal antibodies N19 and C20 (1 : 200 in TBS), after prior antigen retrieval by heating the sections in a pressure cooker in 1 mmol/l ethylenediamine tetraacetic acid buffer, pH 8.0 for 1.5 h. As for CD31 immunostaining, the sections were incubated with biotinylated IgG and then with peroxidase-conjugated streptavidin (LSAB2; DakoCytomation). The colour was developed by diaminobenzidine. The immunodetection of Epo and EpoR was performed with alkaline phosphatase–antialkaline phosphatase (DakoCytomation) and fast red as chromogen, followed by haematoxylin counterstaining. Negative controls included an unrelated monoclonal IgG1 produced by the P3X63/Ag8 mouse secretory myeloma replacing the antibody, for the MAb against CD31^14^ and preincubation with a 10-fold excess of specific blocking peptide (Santa Cruz) for the polyclonal antibodies against Epo and EpoR.

### Microvessel density, Epo and EpoR expression counting

These were simultaneously assessed without knowledge of the final pathological diagnosis by two investigators with a double-headed light microscope (Axioplan II; Zeiss, Oberkochen, Germany). Four to six 200× fields covering almost the whole of each of three sections per sample were examined with a 144-intersection point square reticulum (0.78 mm^2^) inserted in the eyepiece. Care was taken to select microvessels, i.e. capillaries and small venules, from all the CD31-stained vessels. They were identified as transversely sectioned tubes with a single layer of endothelial cells, with or without a thin basement membrane. Each assessment was agreed upon in turn. Microvessels were counted with a planimetric point-count method with slight modifications to restrict counting to transversely cut microvessels occupying the reticulum intersection points.^15^ As the microvessel diameter was smaller than the distance between adjacent points, only one transversely sectioned microvessel could occupy a given point. Microvessels transversely sectioned outside the points and those longitudinally or tangentially sectioned were omitted. Therefore, it was sufficiently certain that a given microvessel was counted only once, even in the presence of several of its section planes. As almost the entire section was analysed per sample and as transversely sectioned microvessels hit the intersection points randomly, the method allowed objective counts. Tumour and endothelial cells stained with anti-CD31, anti-Epo and anti-EpoR antibodies were counted on four to six fields covering the whole of each of three sections adjacent to those stained for microvessels and means ± 1 SD and medians were determined for each section, sample and group of samples. The relationship between microvessel density, Epo and EpoR expression and histopathological type was examined by χ^2^ test or logistic regression analysis. A cut-off value of 10 marked cells was assigned to microvessel density and to EpoR expression in endothelial cells and of 30 marked cells to EpoR expression in tumour cells. Statistical significance was defined as *P* < 0.05.

## Results

**Figure 1:**
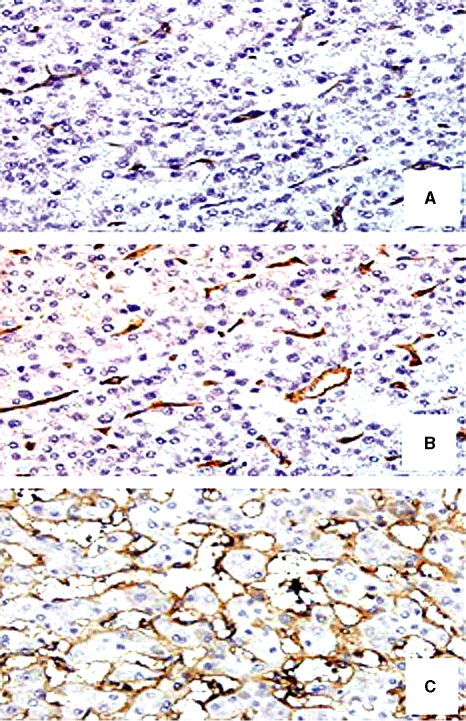
Immunohistochemical reactivity for CD31 in human hepatocellular carcinoma (HCC). Note in (**A**) focal reactivity of a few sinusoids in normal liver; in (**B**) a moderate increase in cirrhotic liver and in (**C**) strong immunoreactivity in a poorly differentiated HCC.

In normal liver, staining with anti-CD31 revealed only focal reactivity of a few sinusoids (Figure 1A). In cirrhotic liver tissue there was a moderate increase in the number of sinusoids stained by anti-CD31 compared with the normal liver (Figure 1B). Moreover, endothelial immunostaining of liver sections with an anti-CD31 antibody showed the formation of tubular-like structures in inflamed portal tracts and septa. Expression of CD31 binding sites was detected in the sinusoidal endothelial cells of cancerous tissues. This expression was stronger in poorly differentiated HCC (Figure 1C) than in well-differentiated HCC. Comparison with adjacent non-cancerous areas showed that this expression was stronger in cancerous areas than in adjacent non-cancerous areas.

**Figure 2:**
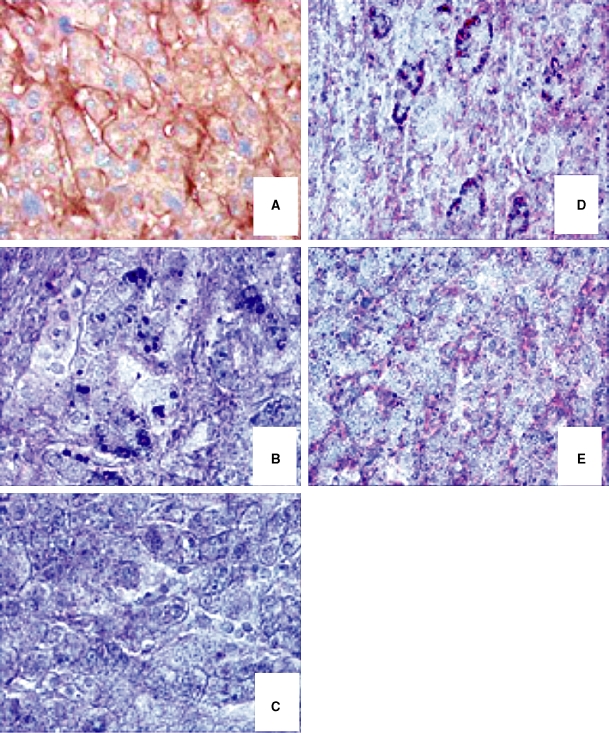
Immunohistochemical reactivity for erythropoietin (Epo) and erythropoietin receptor (EpoR) in human hepatocellular carcinoma (HCC). Note in (**A**) strong immunoreactivity for EpoR in sinusoidal endothelial cells in a poorly differentiated HCC; in (**B**) membrane-linear/cytoplasmic staining for EpoR in tumour cells of a poorly differentiated HCC; in (**C**) absence of staining for EpoR in cirrhotic cells; in (**D**) weak and cytoplasmic granular staining for Epo in tumour cells of a poorly differentiated HCC; in (**E**) absence of staining for Epo in cirrhotic cells.

EpoR was diffusely and strongly expressed on sinusoidal endothelial cells within neoplastic nodules (Figure 2A). A membrane-linear/cytoplasmic immunoreactivity was also noted in a few HCC cells (Figure 2B). A less intense and focal positivity was detected on the sinusoids of the adjacent cirrhotic areas, whereas epithelial cirrhotic cells were negative (Figure 2C). In most cases Epo showed a weak and focal cytoplasmic granular pattern in HCC cells, more prominent in poorly differentiated cases (Figure 2D) than in better differentiated ones; cirrhotic nodules were unstained (Figure 2E).

Table 1 shows the correlation between microvessel, Epo and EpoR counts and histological tumour grade. The comparison of microvessel counts between histological groups revealed statistically significant differences. When differences were sought between groups, significantly higher counts were found in poorly differentiated HCC compared with other stages (*P* < 0.001). Regarding differences in microvessel, Epo and EpoR expression in both endothelial and tumour cells, the within-group comparison showed that both counts were always significantly correlated. There was a trend for these three parameters to increase with histological grade. The counts of patients with poorly differentiated HCC were significantly higher (*P* < 0.001) than those of patients with moderately and well-differentiated HCC, respectively.

**Table 1:** Correlation between histological grade, microvessel density, erythropoietin (Epo) and erythropoietin receptor (EpoR) expression in hepatocellular carcinoma

				EpoR expression	
				
Histological grade	Cases	Microvessel density	Epo expression, tumour cells	Endothelial cells	Tumour cells
Poorly differentiated	14	32 ± 6*	13 ± 4*	27 ± 5*	15 ± 6*
Moderately differentiated	12	20 ± 4*	9 ± 3*	17 ± 4*	10 ± 3*
Well differentiated	24	15 ± 5	5 ± 2	12 ± 4	7 ± 2

* *P* < 0.001 compared with well differentiated.

## Discussion

The results of this study have shown that ‘poorly differentiated’ HCC has a higher degree of vascularization than ‘moderately’ and ‘well-differentiated’ HCC, respectively, and that Epo expression in tumour cells and EpoR expression in both tumour and endothelial cells increases in parallel with the grade of malignancy and is highly correlated with the extent of angiogenesis. Tumour angiogenesis does not depend on a single molecule, since many angiogenic inducers and inhibitors are simultaneously expressed. Secretion by HCC cells, infiltrating inflammatory cells and hepatic stellate cells of several angiogenic cytokines, such as VEGF, FGF-2, angiogenin, angiopoietins and Epo promotes the sprouting of new vessels from pre-existing ones.

The presence of an autocrine-paracrine Epo–EpoR system in tumours and the possible effects of Epo on the tumour microenvironment and angiogenesis are consistent with a complex biology for Epo–EpoR signalling in cancer.

Kayser and Gabius^16^ first suggested that human tumours may express EpoR. In their study 81% of human lung carcinoma tissues possessed Epo-binding sites, detected by the use of biotinylated rhEpo. EpoR transcripts and EpoR protein were subsequently demonstrated in human renal carcinoma,^17^ tumours of the cervix and other organs of the female reproductive tract^18^–^20^ and in various specimens of common paediatric tumours such as neuroblastomas, brain tumours, hepatoblastomas and Wilms' tumours.^21^ By immunohistochemistry, EpoR has been shown to be expressed in breast carcinoma^22^–^24^ and in vestibular schwannoma.^25^ Yasuda *et al*.^26^ studied the expression of Epo in several malignant human cell lines and found that they express Epo and EpoR regardless of their origins, types, genetic characteristics and biological properties, that they secrete a very small amount of Epo individually and that most of them respond to hypoxic stimuli by enhanced secretion of Epo.

There is evidence that HCC produces marked erythrocytosis and is associated with increased levels of serum Epo or the production of Epo-like activity in cell cultures.^27^, ^28^ Because HCC is a hypervascular tumour and the liver and kidney are major Epo production sites, it is suggested that Epo signalling may contribute to the development and progression of malignant hepatic tumours.

Sugimachi *et al*.^29^ investigated Epo expression in a human HCC line and demonstrated that Epo was up-regulated in hypoxic conditions. Nakamatsu *et al*.^30^ investigated the expression of Epo and EpoR in murine chemically induced hepatic tumours. They showed that Epo was not detectable in the normal or cirrhotic liver tissues without tumours, while immunoreactive EpoR was detectable in the endothelium of intervening vessels of all hepatic tumours. No immunoreactive EpoR was discernible in the wall of the hepatic vasculature in the cirrhotic tissues adjacent to the tumours. The reason for this selective expression of EpoR in the tumour vessels is unclear. The immature nature of tumour vessels compared with mature hepatic vessels may be related to the selective expression of EpoR.

These findings suggest the presence of a loop in the Epo–EpoR system, i.e. Epo is secreted by hepatic tumour cells and it affects vascular endothelial cells via its receptors and promotes angiogenesis in a paracrine manner. Thus, it is suggested that Epo is an important factor in hepatic tumour angiogenesis.

Understanding the mechanisms of HCC angiogenesis provides the basis for a rational approach to the development of antiangiogenic therapy in patients with HCC. Inhibition of Epo signalling, by the injection of an anti-Epo monoclonal antibody or a soluble form of EpoR, results in delay of tumour growth in ovarian and uterine cancers.^18^ In nude mice, Yasuda *et al*.^26^ blocked the Epo signalling in xenografts of two representative cell lines by intraperitoneal injections of EpoR antagonist and found inhibition of angiogenesis and survival of tumour cells leading to destruction of tumour masses.

These results may have implications for the treatment of HCC by inhibition of the Epo–EpoR system.

## Abbreviations

CAM: chorioallantoic membrane
Epo: erythropoietin
EpoR: erythropoietin receptor
FGF: fibroblast growth factor
HCC: hepatocellular carcinoma
MAb: monoclonal antibody
VEGF: vascular endothelial growth factor
